# The 125th Anniversary of Aspirin—The Story Continues

**DOI:** 10.3390/ph17040437

**Published:** 2024-03-28

**Authors:** Oliver Werz, Hans Stettler, Christoph Theurer, Jens Seibel

**Affiliations:** 1 Department of Pharmaceutical/Medicinal Chemistry, Institute of Pharmacy, Friedrich Schiller University Jena, 07743 Jena, Germany; oliver.werz@uni-jena.de; 2 Bayer Consumer Care AG, Peter Merian-Strasse 84, 4002 Basel, Switzerland; hans.stettler@bayer.com; 3 Bayer Vital GmbH, Kaiser-Wilhelm-Allee 70, 51373 Leverkusen, Germany; christoph.theurer@bayer.com

**Keywords:** aspirin, acetylsalicylic acid, history, future, antiviral, anniversary

## Abstract

The year 2024 marks the 125th anniversary of aspirin, still one of the most frequently used drugs worldwide. Despite its veritable age, it is still relevant in pharmacotherapy and its use has spread to new areas over time. Due to aspirin’s multiple pharmacological actions unified in one single molecule (i.e., analgesic, antipyretic, anti-inflammatory, antithrombotic, and antiviral effects), it continues to attract considerable attention in the scientific community and is subject to intense basic and clinical research. In fact, recent results confirmed aspirin’s potential role as an antiviral drug and as an agent that can block harmful platelet functions in inflammatory/immunological processes. These features may open up new horizons for this ancient drug. The future of aspirin looks, therefore, bright and promising. Aspirin is not yet ready for retirement; on the contrary, its success story continues. This 125th anniversary paper will concisely review the various therapeutic uses of aspirin with a particular emphasis on the latest research results and their implications (e.g., use as an antiviral agent). In addition, the reader is provided with future perspectives for this remarkable drug.

## 1. Introduction

In 1897, acetylsalicylic acid was first synthesized as a chemically pure and stable compound in a Bayer laboratory in Wuppertal, Germany. Two years later, Bayer launched acetylsalicylic acid in powder form on the market under the trademark name “Aspirin” [1,2]. Specifically, “Aspirin” was entered in the trademark register of the Imperial Patent Office in Berlin, Germany, on 6 March 1899 (No. 36433) [3]. Hence, the year 2024 marks the 125th anniversary of aspirin, still one of the most frequently used drugs worldwide [2,4]. Actually, the history of the discovery of aspirin dates back to more than 3500 years, when the bark of the willow tree was used as a natural remedy to treat pain and fever [4]. Nowadays, various aspirin formulations are available (e.g., tablets, granules, and effervescent forms), designed to meet individual needs. Different routes of administration are also feasible (e.g., oral, intravenous, and buccal) [5].

Following its market introduction, aspirin quickly became widely known as an effective analgesic and antipyretic agent [4]. Later, the anti-inflammatory and antithrombotic properties of aspirin were discovered, paving the way for further therapeutic areas [3,6,7]. Although representing an old drug, aspirin is still relevant and widely used in a wide range of indications. For instance, the antiplatelet effect of aspirin led to its use in the field of cardiology, where it remains the cornerstone of antiplatelet therapy in patients with cardiovascular disease, in both the acute setting as well as for the long-term prevention of cardiovascular events [8,9].

At the time of its 125th anniversary, the success story of aspirin is not over. It continues to attract considerable attention in the scientific community and is subject to intense basic and clinical research. A PubMed search using the keyword “aspirin” yielded more than 20,000 references between 2013 and 2023, reflecting the great interest in the pharmacological and therapeutic potential of aspirin. As an example, recent results from experimental and clinical studies confirmed aspirin’s role as an antiviral agent, which may open up new horizons for this ancient drug [10,11]. This 125th anniversary paper concisely reviews the various therapeutic uses of aspirin with a particular emphasis on the latest research results and their implications (e.g., use as an antiviral agent). In addition, the reader is provided with future perspectives for this remarkable drug.

## 2. Pharmacodynamic Properties of Aspirin—Recent Insights

Aspirin is well known for its analgesic, antipyretic, anti-inflammatory, and antithrombotic actions [5,12]. These pharmacodynamic effects are due to the inhibition of prostaglandin and thromboxane biosynthesis, which is primarily mediated by the irreversible acetylation of specific serine residues in the cyclo-oxygenase-1 (COX-1) and COX-2 isoenzymes. These enzymes are responsible for converting arachidonic acid into prostaglandins, which are lipid mediators involved in inflammation, pain, and fever [13]. While COX-1 is expressed constitutively in many tissues, COX-2 is mainly an inducible enzyme that is upregulated by inflammatory stimuli, hormones, and growth factors [14].

The acetylation of COX-1 leads to a concentration-dependent irreversible inhibition of thromboxane A_2_ production in platelets, a prostanoid that promotes platelet aggregation [15]. The latter is considered the main target of aspirin with regard to its antithrombotic effect. Moreover, aspirin may also act as an antithrombotic agent by other mechanisms, such as the protection of endothelial cells from oxidative stress via the stimulation of the endothelial nitric oxide synthase or by reducing thrombin generation as well as enhancing fibrinolysis [5,16]. In conjunction with lipoxygenases, acetylated COX-2 can generate aspirin-triggered lipoxins, protectins, and resolvins, which are inflammation-resolving lipid mediators that actively terminate inflammation and promote tissue regeneration, potentially contributing to the clinical benefits of aspirin in inflammation-related diseases [17].

Apart from the classical mechanisms of aspirin-induced effects, the drug has a variety of other actions, e.g., the inhibition of the nuclear factor-κB (NF-κB) signaling pathway, the modulation of immune cells, and the blocking of harmful platelet functions in inflammatory/immunological processes [5,18,19,20]. There has been a sustained interest in the discovery of new pharmacological properties of aspirin, most recently its antiviral effects for the prevention and treatment of influenza symptoms and—potentially—severe acute respiratory syndrome coronavirus type 2 (SARS-CoV-2) infections [5]. Triggered by the coronavirus disease-19 (COVID-19) pandemic, the anti-inflammatory and antiviral effects of aspirin regained attention and have become the focus of scientific research. In fact, several in vitro and experimental in vivo studies confirmed the antiviral effects of aspirin against both RNA and DNA viruses (Table 1).

**Figure 1:**
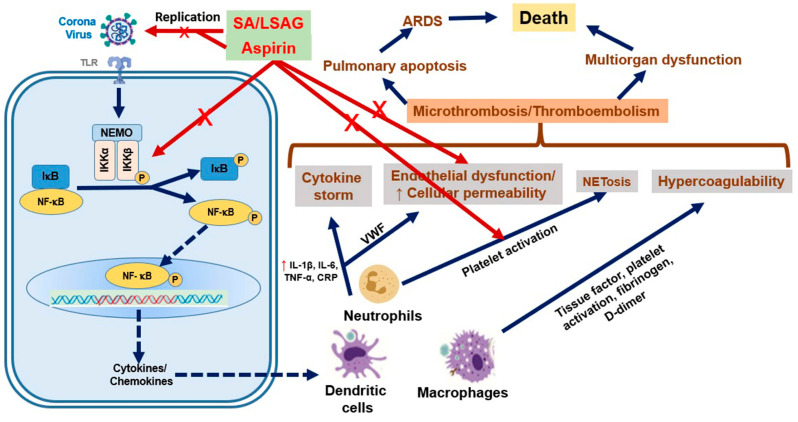
Host cell response to SARS-CoV-2 and the role of aspirin in SARS-CoV-2 infection. SARS-CoV-2-induced cytokine storm, endothelial dysfunction, NETosis, and hypercoagulability result in microthrombosis/thromboembolism in the lungs as well as heart and kidneys, leading to multiorgan dysfunction, ARDS, and ultimately death in a substantial percentage of patients. Aspirin/SA/LASAG can attenuate the viral replication and inhibit NF-κB activation and the subsequent expression of cytokines and chemokines. In addition, ASA exhibits anti-inflammatory and antiplatelet effects and attenuates NETosis, endothelial dysfunction, and hypercoagulability. Abbreviations: ASA, acetyl salicylic acid; SA, salicylic acid; LASAG, D,L-lysine-acetylsalicylate glycine; NF-κB, nuclear factor-κB; IL, interleukin; TNF-α, tumor necrosis factor-α; CRP, C-reactive protein; MCP-1, macrophage chemoattractant protein-1; ICAM-1, intercellular adhesion molecule-1; VCAM-1, vascular cell adhesion molecule-1; NETs, neutrophil extracellular traps; ARDS, acute respiratory distress syndrome. From Ref. [10]. Originally published by and used with permission from Dove Medical Press Ltd.

In most cases, aspirin’s antiviral actions are due to the modulation of virus-induced intracellular signaling pathways in host cells (i.e., a host-directed mechanism of action). Specifically, NF-κB inhibition appears to play a major role [14,37]. However, pathways independent of NF-κB have also been reported for salicylates [40]. The antiviral effects of aspirin complement its clinically proven anti-inflammatory, antithrombotic, and antipyretic actions and may provide additional insights into its mode of action in the relieve of cold and flu symptoms (e.g., pain or fever), an approved indication of aspirin. Due to these pharmacological properties combined in one molecule, aspirin would also be an interesting candidate for an adjunctive treatment of COVID-19 [10]. According to current knowledge, severe SARS-CoV-2 infections are associated with an excessive activation of the immune system, a cytokine storm (hyperinflammation), and a prothrombotic status as expressed by platelet activation, microvascular/macrovascular thrombosis, and multiorgan dysfunction [41]. Hence, aspirin’s anti-inflammatory, antithrombotic, and antiviral effects might have a favorable impact on the clinical course of COVID-19 via the mechanisms summarized in Figure 1 [5,10,41].

## 3. Aspirin as an Anti-Inflammatory, Analgesic, and Antipyretic Agent

### 3.1. Anti-Inflammatory Action—Clinical Experience

Aspirin is the oldest non-steroidal anti-inflammatory drug (NSAID) [42]. While its importance in the treatment of chronic inflammatory joint diseases has decreased over time, its anti-inflammatory properties are still vigorously exploited in severe inflammatory disorders that are associated with an elevated risk of cardiovascular complications, such as Kawasaki disease, adult-onset Still’s disease, rheumatic fever, or recurrent pericarditis [5,14]. Doses ranging from 650 mg to 4 g/day have been used in this setting and led to a significant reduction in inflammation-related symptoms and inflammatory marker concentrations in the plasma [14,43,44,45,46,47,48].

**Figure 2:**
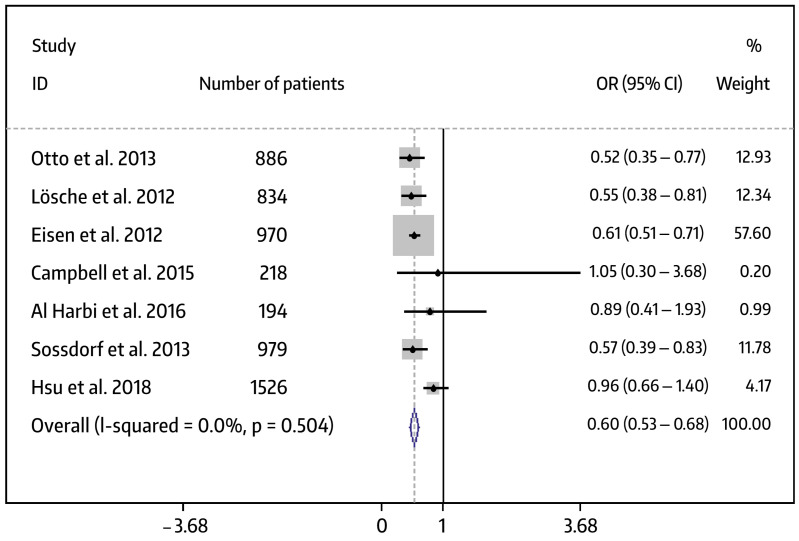
Forest plots showing the effect of aspirin on the mortality rate of patients with sepsis [53,54,55,56,57,58,59]. From Ref. [52] with kind permission from Elsevier (www.elsevier.com).

The more recent discovery that platelets play an important role in inflammatory and immunological processes was the impetus for investigating the role of aspirin in systemic inflammatory diseases, in which platelet activation is a key factor, e.g., sepsis, systemic inflammatory response syndrome (SIRS), or acute respiratory distress syndrome (ARDS). Aspirin is thought to target the immunothrombotic cascade upstream of procoagulant neutrophil extracellular traps (NETs), potentially due to an antiplatelet effect, thereby attenuating infection-induced intravascular coagulation and its associated organ damage [5,49,50]. Therefore, the inhibition of harmful platelet functions by aspirin may be a valuable approach to mitigate the clinical course of sepsis, SIRS, and ARDS. In this context, most clinical trials on sepsis and ARDS conducted over the last decade have produced encouraging results. Ho and colleagues performed a retrospective study in 1,802,034 hospital admissions due to sepsis, of which 195,749 (10.9%) took aspirin. The in-hospital mortality of septic patients on aspirin was lower compared to that of those receiving no aspirin (7.3% vs. 10.1%, respectively; *p* < 0.001), and the length of hospitalization was shorter (6.1 vs. 7.4 days, respectively; *p* < 0.001) [51]. Ouyang and co-workers conducted a meta-analysis on the effect of antiplatelet drugs on the prognosis of sepsis patients [52]. Ten cohort studies were included in the meta-analysis, seven of which used aspirin in a total of 5607 patients. A subgroup analysis revealed that aspirin significantly decreased ICU/hospital mortality in patients suffering from sepsis (odds ratio (OR): 0.60; 95% confidence interval (CI): 0.53–0.68; *p* < 0.05). The associated forest plots are shown in Figure 2.

A previous meta-analysis using propensity matching investigated the association between aspirin use before the onset of sepsis and mortality in hospitalized patients. In total, 6823 sepsis patients were included in the analysis. Taking aspirin was associated with a 7% lower risk of death (95% CI: 2–12%; *p* = 0.005) [60]. Eisen et al. conducted a randomized, placebo-controlled, double-blind, primary prevention study to determine whether low-dose aspirin (100 mg/day) reduces the number of deaths or hospital admissions associated with sepsis in older people, who were healthy at study entry. A total of 16,703 subjects aged ≥70 years were followed up for a median of 4.6 years; 8322 study participants were randomized to receive aspirin and 8381 took a placebo. The use of low-dose aspirin did not lead to a reduction in the mortality rate or the number of hospitalizations associated with sepsis [61]. However, the study only included older people in good health and the number of sepsis-related deaths was low in both groups (1.2% each), rendering it difficult to detect a difference, if there was one.

In regard to ARDS, several meta-analyses have been conducted to investigate the benefits of aspirin for the prevention or treatment of ARDS. Yu et al. analyzed 6562 patients from six trials to determine aspirin’s potential to prevent ARDS and acute lung injury (ALI). It was shown that aspirin could reduce the incidence of ARDS/ALI (OR: 0.71; 95% CI: 0.58–0.86) but not the mortality rate (OR: 0.87; 95% CI: 0.71–1.07) [62]. Panka and colleagues included eight clinical studies in their meta-analysis. The results suggested a beneficial association between aspirin and ARDS prevention [63]. Liang et al. analyzed 6764 at-risk patients from seven studies. The meta-analysis revealed that aspirin was able to reduce the incidence of ARDS (OR: 0.78; 95% CI: 0.64–0.96; *p* = 0.018) but had no significant effect on the in-hospital mortality rate (OR: 0.88; 95% CI: 0.73–1.07; *p* = 0.204) [64]. A randomized double-blind clinical trial investigated whether the early administration of aspirin in adult high-risk patients leads to a lower incidence of ARDS. In total, 400 patients were randomized (1:1) to study groups. In the active group, aspirin was administered as a loading dose (325 mg) on study day 1, followed by 81 mg once daily up to day 7 of the study. The results showed that, in high-risk patients admitted to the emergency department, aspirin use did not significantly reduce the risk of ARDS by study day 7 when compared to the placebo [65]. However, this study had several limitations, such as an unanticipated low ARDS rate [66]. A further randomized double-blind study was performed to determine whether aspirin at 75 mg/day is effective in improving the oxygenation index in hospitalized ARDS patients at study day 7. Aspirin (*n* = 24) did not improve the oxygenation index when compared to the placebo (*n* = 25). However, the study had to be terminated prematurely due to slow recruitment [67].

It has been postulated that a cocktail therapy, in which aspirin is combined with certain drugs, could be favorable for the prevention/treatment of ARDS [66].

### 3.2. Analgesic Action—Clinical Experience

Numerous clinical studies and meta-analyses confirmed that aspirin at a standard single dose ranging from 0.5 to 1 g is an effective and safe analgesic for the treatment of various types of acute to moderately severe pain, including sore throat, tension-type headache, migraine, and dental pain [5]. Also in the postoperative setting, aspirin proved to be an effective analgesic [68,69]. The analgesic effect augments with the increase in the dose, but high doses are accompanied by an increased incidence of adverse effects (e.g., gastric irritation) [5,69,70]. The pain relief achieved with aspirin is comparable to that of an equivalent dose of paracetamol (=acetaminophen) [71,72].

**Figure 3:**
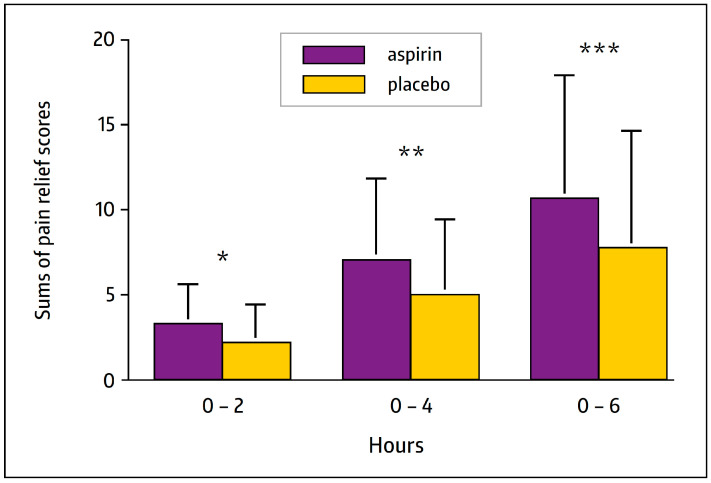
Sums of pain relief scores for sore throat at 2, 4, and 6 h after treatment. *p*-values are for comparisons between the treatment groups for each time period: * *p* < 0.0001, ** *p* = 0.0002, and *** *p* = 0.0006. Modified from Ref. [73].

Aspirin is frequently used for the treatment of symptoms related to both common cold and influenza, including pain symptoms. In a randomized, placebo-controlled, double-blind study in 272 patients with sore throat pain and other pain symptoms associated with acute upper respiratory tract infection (URTI), a single dose of 800 mg aspirin (effervescent tablet) provided effective relief from sore throat pain, headache, muscle pain, and other URTI-related pain, and was significantly better than the placebo on these endpoints, as exemplified in Figure 3 [73].

**Figure 4:**
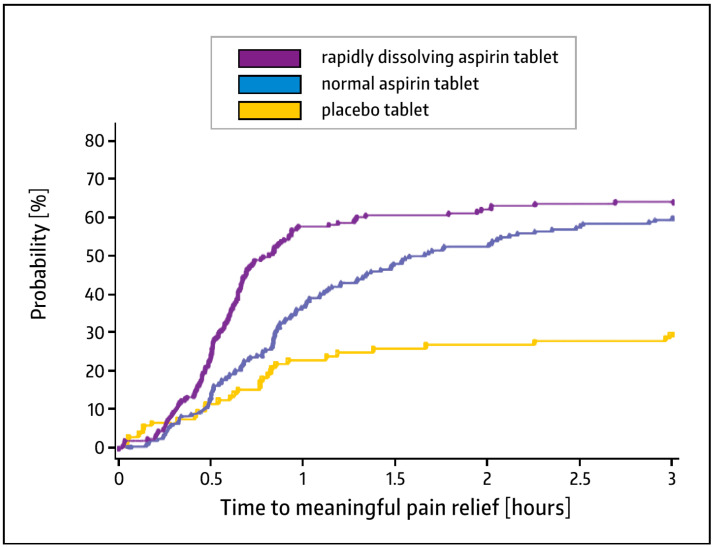
Kaplan–Meier plot of time to meaningful pain relief from aspirin 500 mg. Modified from Ref. [75].

For pharmacokinetic reasons, pre-dissolved, water-soluble, or fast disintegrating aspirin formulations are preferable, as a faster onset of action and an initially higher efficacy in terms of pain relief is achieved [5]. The dental impaction pain model is often used to determine the analgesic effect of analgesics and is well established [74]. Cooper and Voelker used this model to compare a rapidly dissolving swallowable tablet (containing micronized aspirin) to regular aspirin tablets and a placebo. It was shown that the time to meaningful pain relief was significantly shorter with micronized aspirin; both aspirin formulations performed significantly better than the placebo (Figure 4) [75].

Useful combinations with aspirin as an analgesic include pseudoephedrine, caffeine, and metoclopramide when used for the treatment of URTI, headache, and migraine attacks, respectively [5,76,77,78,79].

### 3.3. Antipyretic Action—Clinical Experience

Aspirin is an effective antipyretic analgesic and is often used for treating fever associated with colds and influenza-like illnesses. The antipyretic effect of aspirin is dose-dependent [5].

**Figure 5:**
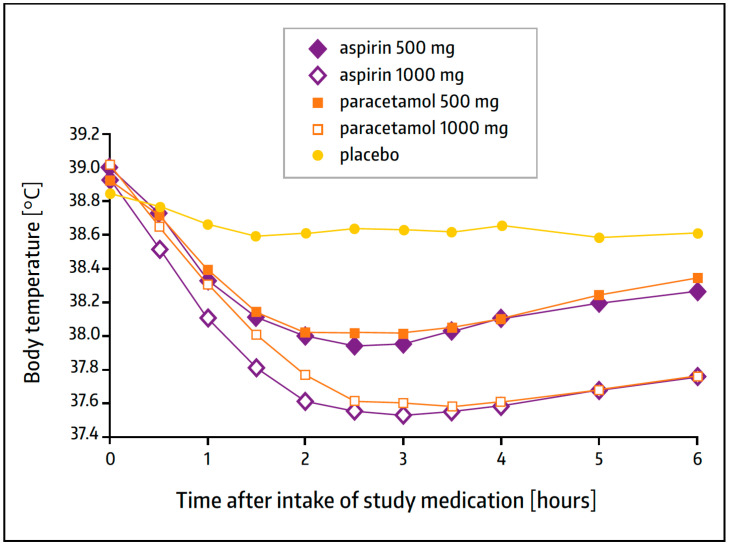
Time course of orally measured body temperature (mean values) after treatment with aspirin, paracetamol, or a placebo. Modified from Ref. [71].

Bachert and colleagues conducted a randomized, double-blind, placebo-controlled study in a total of 392 adult patients with acute febrile URTI (≥38.5 °C) of presumed viral cause [71]. The study participants received a single dose of aspirin 500 or 1000 mg, paracetamol 500 or 1000 mg, or the corresponding placebo. The effect on fever was determined at various times during the first six hours. Both active treatments reduced fever in a dose-related manner and were significantly superior to the placebo. With aspirin, the maximum reduction in fever was observed at 2.5–3 h following drug intake, but the effect started to begin after 30 min and lasted throughout the observation period (Figure 5). There was no significant difference between paracetamol and aspirin.

The antipyretic effect of aspirin matches ideally with the potential antiviral effects of aspirin and represents a useful pharmacodynamic combination.

## 4. Aspirin as an Antiplatelet/Antithrombotic Agent

The daily administration of low doses of aspirin (e.g., 75–100 mg/day) leads to an inhibition of aspirin-sensitive platelet functions within a few days and thus to an antithrombotic action [80,81,82]. Aspirin’s antiplatelet effect is irreversible and can only be offset by new platelets coming from the bone marrow [5]. The efficacy of low-dose aspirin as an antiplatelet drug has been demonstrated in various randomized controlled trials, as recently reviewed by C. Patrono [9], with higher doses not being more effective than lower doses in terms of antithrombotic action. Against this background, low-dose aspirin has been used successfully for the inhibition of platelet aggregation and the prevention of ischemic cardiovascular events, such as myocardial infarction or stroke [1,14], with sex differences in the efficacy, depending on the event [83]. Still, today, low-dose aspirin is the cornerstone of antithrombotic therapy [9]. For instance, the 2021 guideline of the European Society of Cardiology recommends low-dose aspirin (75–100 mg/day) as the drug of first choice for the secondary prevention of cardiovascular events in patients with a previous myocardial infarction or revascularization (evidence level IA) [84]. Aspirin’s role in primary prevention is still controversially discussed [5].

**Figure 6:**
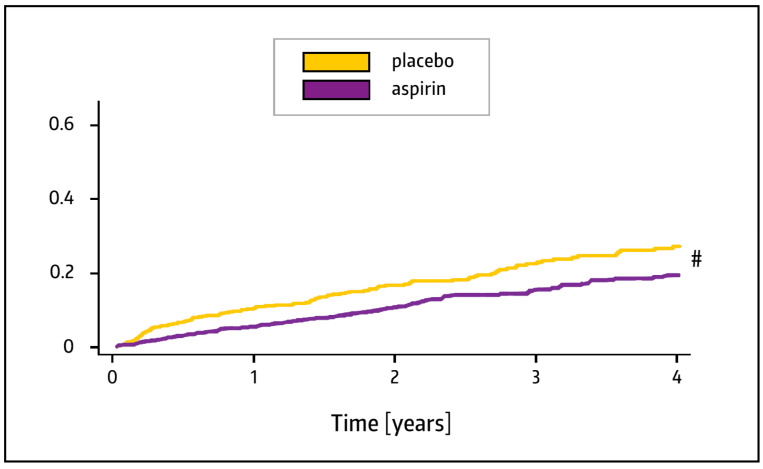
Cumulative risk of venous thromboembolism in the years following randomization, defined as symptomatic deep-vein thrombosis or pulmonary embolism. ^#^ Hazard ratio: 0.68; 95% CI: 0.51–0.90; *p* = 0.008. Modified from Ref. [87].

The prospectively planned combined analysis of the Warfarin and Aspirin (WARFASA) study [85] and the Aspirin to Prevent Recurrent Venous Thromboembolism (ASPIRE) study [86] revealed that aspirin (100 mg/day) safely reduced the rate of thrombotic event recurrence in patients with a first unprovoked venous thromboembolism who had completed initial treatment with heparin/warfarin or an equivalent anticoagulant regimen. Specifically, aspirin significantly reduced the risk by 32% compared to the placebo (Figure 6) [87]. The rate of bleeding events was low and did not differ significantly between study groups (0.7% per year for the placebo and 1.1% per year for aspirin) [87] (see also Section 7). These data suggest that platelets contribute to the initiation and progression of venous thromboembolism and indicate that aspirin is not only able to prevent arterial thrombosis, but also moderately reduces venous thromboembolism [88,89]. More recent studies and meta-analyses support this assumption [90,91,92], with the caveat that non-vitamin K antagonist oral anticoagulants also play a crucial role in this setting [93]. Nevertheless, a drug with a dual preventive action on venous and arterial thrombosis would be welcome in patients with an increased risk for both (e.g., infectious disease patients with an increased risk of disseminated intravascular coagulation or infection-induced coagulopathy) [14]. Recently, there has been a call for simple strategies to reduce COVID-19-associated arterial thromboses and venous thromboembolic events [94]. The administration of aspirin could be a valuable option for patients who are in a prothrombotic state caused by SARS-CoV-2.

## 5. Aspirin and Tumor Prevention

Most data are available for tumors of the gastrointestinal tract (i.e., colon cancer and adenomas). In 1988, Kune and co-workers published the first trial on the use of aspirin and colon cancer prevention. According to their retrospective analysis, the continued daily intake of aspirin was associated with an approximately 40% reduced risk for colorectal cancer development [95]. Since then, large observational trials and several randomized clinical studies essentially corroborated this initial finding [96]. In addition, various studies indicated that aspirin has the potential to delay the metastatic dissemination of cancer with a prolongation of patient survival [97]. The results from recent meta-analyses of 118 observational studies in patients with 18 different cancers suggested that there is an approximately 20% mortality reduction upon long-term intake of aspirin (Table 2). The authors, therefore, proposed that aspirin should be considered seriously as an adjuvant in cancer treatment, and that cancer patients and their caregivers should be informed about the current evidence [96,98]. In fact, due to its capability to irreversibly inhibit platelet activation, thereby blocking platelet-mediated tumor growth and multiple steps of its progression, low-dose aspirin may be an effective adjuvant to be administered in combination with established chemotherapeutic regimens in the oncologic setting [97,99,100].

However, there are inconsistencies in study results and there is a lack of evidence for a lower mortality rate from the randomized controlled trials of aspirin. Moreover, a specific group of patients based on age and weight/body seize appear not to respond to aspirin’s anti-cancer effects, for reasons that are currently unknown [97,101]. The Aspirin in Reducing Events in the Elderly (ASPREE) trial was a large randomized, placebo-controlled primary prevention study in subjects with advanced age who did not have cardiovascular disease, dementia, or disability. Despite several study limitations, the principal finding remained that aspirin apparently had no favorable effects in the population studied and that, in older persons, aspirin may even promote cancer progression [102,103,104]. This unexpected observation needs be elucidated further.

Extended evidence for a protective effect of aspirin against the development of colorectal and other cancers is expected in the next few years from ongoing large clinical studies in which aspirin is used at low dosages over a long term in the primary or secondary prevention setting [9,100,105].

## 6. Aspirin and Infections

The multifaceted pharmacological actions of aspirin render it a promising candidate for an adjunctive use in infectious diseases. As lately reviewed by Di Bella and colleagues, aspirin has intrinsic anti-infective and anti-biofilm activities, mainly demonstrated by preclinical studies. In addition to the beneficial clinical results assessed in sepsis and ARDS patients (see Section 3.1), it is expected that aspirin will be investigated further to identify infectious disease conditions in which it is used as an adjunct drug [41]. Encouraged by several in vitro and experimental in vivo studies (Table 1), particular attention was placed on the supplemental antiviral effects of aspirin in the clinical setting. In fact, due to its unique combined anti-inflammatory, antithrombotic, and antiviral effects, there is a strong rationale for a potential role of aspirin in infections caused by viral pathogens [10,14].

A couple of clinical trials investigated the effects of aspirin in subjects with human immunodeficiency virus (HIV) infection. In a randomized, placebo-controlled, crossover study in HIV-infected patients with abacavir-related platelet hyperreactivity, low-dose aspirin significantly reduced in vivo platelet activation and platelet hyperreactivity, however, without reaching the values of the healthy controls [106]. O’Brien and co-workers showed in a pilot study that one week of low-dose aspirin attenuated markers for platelet and immune activation in HIV-infected adults on antiretroviral therapy [107]. Subsequently, the same group performed a randomized, placebo-controlled trial to investigate the effects of aspirin on the markers of immune activation in HIV-infected adults on suppressive antiretroviral therapy. There was no significant difference to placebo in immune activation markers following 12-week treatments with aspirin (100 mg and 300 mg) [108]. Conversely, a recent randomized study showed that the daily intake of low-dose aspirin for nine weeks was associated with a significant reduction in biomarkers for HIV-associated inflammation in patients with HIV receiving antiretroviral therapy as well as in elite controllers [109].

Aspirin is often used for treating symptoms associated with colds and influenza-like illnesses. In fact, randomized, placebo-controlled clinical studies showed that aspirin significantly attenuated the symptoms of acute URTI of presumed viral cause in comparison to a placebo (Figure 3 and Table 3) [71,73].

The various pharmacological actions of aspirin (i.e., analgesic, antipyretic, anti-inflammatory, antithrombotic, and antiviral effects) positioned the drug as an ideal candidate for the supportive therapy of COVID-19 [10]. In fact, the usefulness of aspirin in this setting has been investigated in some observational studies, randomized controlled trials, meta-analyses, and reviews with inconsistent results, e.g., [10,14,41,110].

By now, a total of six meta-analyses have been published (Table 4). Generally, the meta-analyses concluded that the use of aspirin was significantly associated with a lower risk of death compared to the patients who did not receive the drug. The most recent meta-analysis was performed by Ma and colleagues, which included 17 studies with 49,041 patients. There was a significantly reduced mortality risk in aspirin users relative to non-users (adjusted relative risk (RR): 0.69; 95% CI: 0.50–0.95; *p* < 0.001) in patients hospitalized for COVID-19. A subgroup analysis restricted to those who took low-dose aspirin (80–100 mg/day) also showed a significantly lower risk of a fatal outcome (RR: 0.64; 95% CI: 0.48–0.85; *p* < 0.01) [111]. It should be taken into account that the conclusions of the meta-analyses are limited because most of the included studies followed a retrospective study design [41].

Lately, results from prospective, randomized controlled clinical trials became available. Conners et al. investigated symptomatic but clinically stable outpatients with COVID-19; they were treated with aspirin (81 mg/day) in comparison to a placebo. Regrettably, the trial had to be stopped prematurely due to an unexpected low event rate [117]. In the Randomized Evaluation of COVID-19 Therapy (RECOVERY) study, hospitalized COVID-19 patients were randomized to receive either the standard of care plus aspirin at 150 mg/day until hospital discharge (*n* = 7351) or the standard of care alone (*n* = 7541). The patients in the aspirin group did not show a significantly lower 28-day mortality rate, although a slightly higher proportion of patients was released from hospital alive within 28 days (75% vs. 74%, respectively; rate ratio: 1.06; 95% CI: 1.02–1.10; *p* = 0.006). The study participants randomized to aspirin experienced a slightly reduced median hospitalization time (8 vs. 9 days). As expected, the intake of aspirin was associated with a lower risk of thrombotic events (4.6% vs. 5.3%) and a marginally increased risk of major bleeding events (1.6% vs. 1.0%) [118]. Similarly, results from the randomized controlled Anti-Coronavirus Therapies (ACT) outpatient study provided no supporting evidence for the use of aspirin to prevent disease progression or death in high-risk outpatients with COVID-19, with the caveat that the event rates were lower than expected. In the latter study, patients receiving aspirin at 100 mg/day for 28 days (*n* = 1945) were compared against patients receiving the standard of care (*n* = 1936) [119]. In the context of the still ongoing Randomized, Embedded, Multifactorial Adaptive Platform Trial for Community-Acquired Pneumonia (REMAP-CAP), critically ill adult patients hospitalized for COVID-19 were randomized to receive either aspirin at 75–100 mg/day (*n* = 565) or no antiplatelet therapy (*n* = 529), both in addition to anticoagulation thromboprophylaxis. The median duration of aspirin therapy was twelve days. There was a 96.0% probability that aspirin therapy improved survival to hospital discharge, with an adjusted absolute reduction in mortality of 5.4% (95% credible interval: −0.7–10.5). Major bleeding events occurred at low rates (2.0% and 0.4% of patients in the aspirin and control groups, respectively) [120]. In conclusion, the presently available data are not conclusive regarding the benefits of aspirin in the adjuvant treatment of COVID-19. The timing of aspirin commencement, dosage, treatment duration, and the subgroups of COVID-19 patients who may profit the greatest from aspirin’s pharmacological effects have yet to be determined in the context of further randomized controlled trials [14].

Overall, the results assessed in the clinical setting suggest that aspirin has the potential to provide benefits when used as a companion drug in the treatment of infectious diseases caused by viral pathogens, including possibly COVID-19. Further randomized controlled studies appear to be warranted.

## 7. Safety of Aspirin

While aspirin has various therapeutic effects, its use is associated with potential adverse effects, particularly gastrointestinal complaints ranging from gastritis to gastrointestinal bleeding [12]. In fact, the risk of increased bleeding is considered the most important adverse event of aspirin [16].

Many adverse reactions following the ingestion of aspirin are dose-related and/or occur upon long-term use. Therefore, concerns about the use of aspirin mainly relate to situations in which aspirin is used at high doses and/or over a prolonged period of time [14]. In the long-term administration of aspirin at medium and high doses for the treatment of rheumatic/immunological diseases, adverse effects were observed that were mainly gastrointestinal in nature (e.g., epigastric pain, epigastric discomfort, nausea, or bleeding events). Serious bleedings, such as gastrointestinal tract hemorrhages or cerebral hemorrhages, occurred rarely [5,14]. The long-term use of low aspirin doses (e.g., in cardiocoronary prevention) is considered safe with a very favorable benefit/risk ratio. However, bleeding and other adverse reactions are of concern in particular life situations, such as late pregnancy and advanced age [5]. It has been shown that the risk of severe bleeding events is increased in older patients compared to younger people; concomitant treatment with antithrombotic drugs is also a potential risk factor for the development of gastrointestinal complications during aspirin exposure [121]. This risk can be markedly lowered by the concomitant intake of H_2_-receptor antagonists or proton-pump inhibitors [122]. When initiating aspirin administration during pregnancy, platelet counts and gestational age need to be considered [5,14]. The drug should not be taken in the last trimester of pregnancy. In the general population, the short-term or single use of high aspirin doses (e.g., to treat symptoms associated with colds and influenza-like illnesses) is generally regarded as safe [123,124], but caution should be taken in people at an increased risk of adverse effects, particularly bleeding.

There are also adverse reactions of aspirin that are presumably unrelated to dose. These include the occurrence of bronchospasm, asthma attacks, or other hypersensitivity reactions. The Reye syndrome does also belong to this category [5]. The latter is typically observed in children and represents a rare but life-threatening acute, non-inflammatory encephalopathy with fatty liver failure [125]. A possible association between Reye syndrome and aspirin use of has been suggested but not established. In this context, it must be mentioned that, already in 1980, the Food and Drug Administration and the Centers for Disease Control and Prevention recommended that aspirin should not be used to treat acute febrile viral illness in children and adolescents under 19 years of age, as its use may cause the Reye syndrome [41,125].

In summary, the safety profile of aspirin has been well established and is well known. It is a remarkably safe drug when administered circumspectly [5,126]. Caution must be exercised in people at an increased risk of adverse reactions.

## 8. Aspirin Nowadays

Aspirin is still one of the most frequently used drugs worldwide. Its use in the treatment of cold and flu symptoms remains a domain of aspirin. In addition, aspirin still is the cornerstone of antiplatelet therapy for the acute treatment and long-term prevention of atherothrombosis [9]. Due to aspirin’s multifaceted pharmacological actions, it continues to attract considerable attention in the scientific community and is subject to intense basic and clinical research. Thanks to aspirin, the knowledge of the diverse functions of platelets has expanded considerably [9]. The latter was the impetus for investigating the role of aspirin in systemic inflammatory diseases, in which platelet activation is a key factor, such as sepsis, SIRS, or ARDS. Most clinical trials in this setting have produced encouraging results, but further appropriately sized randomized controlled trials are required. Encouraged by several in vitro and experimental in vivo studies, particular attention was placed on the supplemental antiviral effects of aspirin in the clinical setting. Consequently, aspirin is also being investigated as a possible adjunct in the treatment of HIV and especially COVID-19. In fact, due to its unique combined anti-inflammatory, antithrombotic, and antiviral effects, there is a strong rationale for a potential role of aspirin in infections caused by viral pathogens. Further randomized controlled trials appear to be warranted to test whether aspirin can provide an added benefit to patients suffering from such infections. Finally, another major topic in clinical research is the possible protective effect of aspirin against the development of colorectal and other cancers [5].

## 9. Future Perspectives for Aspirin

Looking ahead, it can be expected that aspirin will continue to play a major role in the field of cardiovascular and cerebrovascular disease prevention. Its role in primary prevention could be reassessed by analyzing individual patient data using meta-analysis techniques [8]. Similarly, its established use in the symptomatic treatment of cold and flu complaints will remain an undisputed domain of aspirin. Apart from that, recent research findings may open up new horizons for this well-known agent. Results from ongoing/planned randomized controlled studies are eagerly awaited to confirm clinically the growing evidence that aspirin has pharmacological features that are highly welcome. Due to its unique combined anti-inflammatory, antithrombotic, and antiviral effects, there is a strong rationale for a potential role of aspirin in infections caused by viral pathogens, making it an ideal adjunctive therapeutic option in the treatment of COVID-19 and other viral infections. Moreover, there is some likelihood that aspirin—in combination with other drugs—will provide benefits to patients suffering from sepsis, SIRS, or ARDS. Extended evidence for a protective effect of aspirin against the development of colorectal and other cancers is expected in the next few years from ongoing large clinical studies. It is becoming increasingly obvious that an individualized approach is important in this case. Depending on the study results, aspirin could become a valuable companion drug in this setting. Future high-quality studies may also clarify whether aspirin has neuroprotective effects, i.e., the capability to prevent/delay neurological diseases with cognitive deficits, such as dementia or Alzheimer’s disease [127]. Finally, it needs to be emphasized that there are multiple ongoing research activities related to aspirin (e.g., effects on gene regulation and transcription) [5]. Given the surprises we have experienced with this remarkable drug in recent decades, further avenues might therefore be pursued as new research results become available.

## 10. Conclusions

Aspirin is widely used in various indications. Despite its veritable age, it is still relevant in pharmacotherapy and its use has spread to new areas over time. At the time of its 125th anniversary, aspirin continues to be subject to intense basic and clinical research. Recent results from preclinical and clinical studies confirmed aspirin’s potential role as an antiviral drug and as an agent that can block harmful platelet functions in inflammatory/immunological processes. Aspirin’s multifaceted pharmacological actions open up new horizons for this ancient drug. The future of aspirin looks bright and promising and awaits the completion/initiation of appropriately sized randomized controlled trials. Aspirin is not yet ready for retirement; on the contrary, its success story continues.

## Figures and Tables

**Table 1:** Summary of the most relevant in vitro and experimental in vivo studies on aspirin’s antiviral actions.

Reference and Type of Study	Virus	Infection Model	Key Results of Aspirin Exposure
[21] In vitro	Influenza viruses (A/H7/N1 and A/H1N1/6/86)	MDCK cells	Inhibition of viral activity
[22] In vitro	RSV	A549 (human lung carci-noma epithelial cells)	Inhibition of RSV-mediated transcriptional induction of multiple cytokines
[23] In vitro	CMV	Coronary artery smooth muscle cells	Inhibition of CMV replication
[24] In vitro	VZV	Human embryonic lung cells	Reduction in VZV replication; the inhibition was partially reversible, depending on the concentration and exposure time
[25] In vitro	VZV	MRC-5 (human lung fibroblast) and Vero (monkey kidney epithelial) cells	High doses reduced VZV replication
[26] In vitro	JEV	N18 (mouse neuroblastoma cells)	Suppression of JEV propagation
[27] In vitro	EBV	EBV-positive B95.8 and Raji cells as well as EBV-negative BJAB cells	Induction of EBV lytic replication in host cells, which resulted in the killing of EBV-positive cells
[28] In vitro	HCV	Huh7 (human hepatoma) cells expressing nonstructural HCV proteins	Suppressive effects on HCV-RNA and protein expression partly caused by inhibiting COX-2 signaling pathways
[29] In vitro	HCV	Huh7 (human hepatoma) cells expressing nonstructural HCV proteins	Reduction in cellular oxidative stress and modulation of Cu/Zn SOD1 expression, thereby reducing the pathogenic effects of HCV
[30] In vitro	HCV	Huh7 (human hepatoma) cells expressing nonstructural HCV proteins	Overexpression of proteins that inhibit HCV replication
[31] In vitro	HCV	Huh7 (human hepatoma) cells expressing nonstructural HCV proteins	Reduction in iNOS expression and HCV-RNA replication
[32] In vitro	HCV	Huh7.5.1 (human hepatoma) cells	Downregulation of claudin-1 expression, an HCV receptor, thereby inhibiting HCV cell entry
[33] In vitro	Zika virus	Zika virus-sensitive cell lines, such as A549 (human lung carcinoma epithelial) and Vero (monkey kidney epithelial) cells	Inhibition of Zika virus replication by downregulating the viral entry cofactor AXL, a receptor tyrosine kinase known to regulate diverse cellular processes
[34] In vitro	Various respiratory RNA viruses, including influenza A H1N1	MDCK, human epithelial, HeLa, and buffalo green monkey cells	Specific antiviral activity against influenza A virus, human rhinoviruses, and coxsackievirus (subtype 9)
[35] In vitro	Human rhinovirus type 14	HeLa cells	Dose-dependent antiviral activity; the antiviral effect involved the suppression of VP3 expression, a major structural protein, and the infection-dependent downregulation of CD54
[11] In vitro	SARS-CoV-2	A549-ACE2 (human lung carcinoma epithelial cells expressing ACE2) as well as Huh7 (human hepatoma) and Vero (monkey kidney epithelial) cells Human precision-cut lung slices	Suppression of SARS-CoV-2 replication by up to two orders of magnitude; a lower viral RNA expression was observed in both models, indicating that post-entry pathways were inhibited
[36] In vitro	Bunyamwera	Vero (monkey kidney epi-thelial) cells	Inhibition of Bunyamwera infections by interfering with the biogenesis of its replication organelle
[37] In vitro and in vivo	Influenza viruses	MDCK and A549 (human lung carcinoma epithelial) cells Mice	Blocking of influenza virus replication in vitro and in vivo via NF-κB inhibition
[38] In vitro and in vivo	Rotavirus	MA104 (rhesus monkey kidney), Caco-2 (human colon adenocarci-noma), and CV-1 (fetal African green monkey kidney) cells Rats	Inhibition of rotavirus infection in cell lines and in rats Alteration of rat gut microbial composition
[39] In vivo	Several types of viruses	Drosophila	Higher resistances to viral infections largely through the mediation of the stimulator of interferon genes signaling pathway

ACE2: angiotensin-converting enzyme 2; CMV: cytomegalovirus; EBV: Epstein–Barr virus; HeLa: human cervical cancer cells; HCV: hepatitis C virus; iNOS: inducible nitric oxide synthase; JEV: Japanese encephalitis virus; MDCK: Madin–Darby canine kidney; RSV: respiratory syncytial virus; SARS-CoV-2: severe acute respiratory syndrome coronavirus type 2; SOD1: superoxide dismutase type 1; VZV: varicella zoster virus.

**Table 2:** Aspirin intake and mortality: meta-analysis of 118 published observational studies *.

Group	Cancer Mortality		All-Cause Mortality	
	No. of Studies	HRs (95% CIs) ORs (95% CIs)	No. of Studies	HRs (95% CIs) ORs (95% CIs)
Colon cancer	24	HR 0.71 (0.62, 0.80)	21	HR 0.81 (0.73, 0.91)
	1	OR 0.78 (0.66, 0.93)	1	OR 0.78 (0.65, 0.92)
Breast cancer	13	HR 0.84 (0.72, 0.98)	9	HR 0.94 (0.70, 1.25)
	4	OR 0.75 (0.36, 1.57)	None	-
Prostate cancer	15	HR 0.89 (0.78, 1.02)	6	HR 1.00 (0.78, 1.27)
	1	OR 1.02 (0.78, 1.34)	1	OR 1.06 (0.94, 1.19)
15 other cancers	18	HR 0.79 (0.70, 0.83)	21	HR 0.67 (0.60, 0.75)
	5	OR 0.49 (0.26, 0.95)	5	OR 0.47 (0.26, 0.83)
All 18 cancers	70	HR 0.77 (0.72, 0.83)	56	HR 0.79 (0.74, 0.86)
	11	OR 0.67 (0.45, 1.00)	7	OR 0.57 (0.36, 0.89)

CIs: confidence intervals; HRs: hazard ratios; ORs: odds ratios. * data from Refs. [96,98].

**Table 3:** Intensity of the selected symptoms of upper respiratory tract infection (aspirin vs. a placebo), rated by patients on a scale from 0 = none to 10 = severe *.

Symptom	Time Point (h)	Aspirin		Placebo
		500 mg	1000 mg	
Headache	0	6.44 (2.10)	6.60 (2.05)	6.12 (2.12)
	2	4.36 (1.94) ^#^	4.00 (1.85) ^#^	5.72 (1.93)
	4	4.03 (1.99) ^#^	3.58 (2.01) ^#^	5.76 (2.14)
	6	4.41 (2.18) ^#^	3.76 (2.26) ^#^	5.78 (2.06)
Achiness	0	6.12 (1.94)	6.21 (2.37)	5.62 (2.25)
	2	4.60 (1.85) ^$^	3.65 (2.11) ^#^	5.36 (2.06)
	4	4.31 (1.97) ^$^	3.19 (2.25) ^#^	5.33 (2.21)
	6	4.77 (2.04)	3.36 (2.47) ^#^	5.26 (2.20)
Feverish discomfort	0	6.96 (1.43)	7.14 (1.74)	6.53 (1.54)
	2	5.00 (1.77) ^#^	4.12 (2.23) ^#^	6.21 (1.75)
	4	4.75 (1.90) ^#^	3.49 (2.36) ^#^	6.08 (2.01)
	6	5.29 (2.04) ^§^	3.71 (2.56) ^#^	6.00 (1.93)

Values are means (SD). ^#^ *p* < 0.001 vs. placebo, 1-sided *t*-test; ^§^ *p* < 0.025 vs. placebo, 1-sided *t*-test; ^$^ *p* < 0.01 vs. placebo, 1-sided *t*-test; * adapted from Ref. [71].

**Table 4:** Meta-analyses investigating the effects of aspirin on the mortality of COVID-19 patients.

Reference	Study Population	Number of Studies/Patients	Key Results	Comment
[112]	Patients with confirmed COVID-19	3 studies/ *n* = 1054	Mortality among aspirin users was 22.6% vs. 18.3% among non-aspirin users (RR: 1.12; 95% CI: 0.84–1.50)	No association was seen between aspirin use and mortality
[113]	Hospitalized COVID-19 patients	6 studies/ *n* = 14,377	Significantly reduced mortality risk in aspirin users relative to non-users (OR: 0.50; 95% CI: 0.32–0.77; HR: 0.50; 95% CI: 0.36–0.69)	Aspirin was associated with a reduced mortality risk but not antiplatelet drugs as a class
[114]	Adult patients with confirmed COVID-19	10 studies/ *n* = 56,696	Significantly reduced mortality risk in aspirin users relative to non-users (OR: 0.70; 95% CI: 0.63–0.77)	No significant effect was seen after the exclusion of outlier studies in terms of sample size
[115]	Hospitalized, adult COVID-19 patients	7 studies/ *n* = 34,415	Significantly reduced mortality risk in aspirin users relative to non-users (RR: 0.56; 95% CI: 0.38–0.81; *p* = 0.002)	Effect of aspirin on the incidence of thrombosis and bleeding could not be judged due to low reporting in the studies
[116]	Hospitalized COVID-19 patients with low-dose aspirin (75–325 mg/day) before or during hospital stay	6 studies/ *n* = 13,993	Significantly reduced mortality risk in aspirin users relative to non-users (RR: 0.46; 95% CI: 0.35–0.61; *p* < 0.001)	Subgroup analysis on in-hospital aspirin use also revealed a significant mortality reduction
[111]	Hospitalized COVID-19 patients	17 studies/ *n* = 49,041	Significantly reduced mortality risk in aspirin users relative to non-users (adjusted RR: 0.69; 95% CI: 0.50–0.95; *p* < 0.001)	Subgroup analysis of low-dose aspirin use (80–100 mg/day) also showed a significant mortality reduction

CI: confidence interval; HR: hazard ratio; *n*: number of patients included in the meta-analysis; OR: odds ratio; RR: relative risk.

## Data Availability

Data sharing is not applicable.
